# Dual therapy of rosiglitazone/pioglitazone with glimepiride on diabetic nephropathy in experimentally induced type 2 diabetes rats

**DOI:** 10.1016/S1674-8301(11)60054-7

**Published:** 2011-11

**Authors:** Ravi Prakash Rao, Ansima Singh, Arun K Jain, Bhartu Parsharthi Srinivasan

**Affiliations:** a Department of Pharmacology, Delhi Institute of Pharmaceutical Sciences and Research (DIPSAR), New Delhi 110017, India;; b Department of Pharmaceutical Science, Birla Institute of Technology, Mesra, Ranchi 201301, India;; c Institute of Pathology, Indian Council of Medical Research (ICMR), New Delhi 110029, India.

**Keywords:** type 2 diabetes mellitus, diabetic nephropathy, peroxisome proliferator activated receptors (PPARs), transforming growth factor-β1 (TGF-β1), tumor necrosis factor-α (TNF-α)

## Abstract

Diabetic nephropathy is a major cause of end-stage renal disease (ESRD) in the general population. It is estimated that diabetic nephropathy will eventually develop in about 40% of all patients with diabetes; therefore, prevention is critical for delaying the development and progression of diabetic kidney disease. Despite extensive efforts, medical advances are still not successful enough to prevent the progression of the disease. In the present study, we focused on the comparison of combination therapies and whether they offered additional renoprotection. Type 2 diabetes mellitus was induced by intraperitoneally administering streptozotocin (90 mg/kg) in neonatal rats and then these rats were treated with rosiglitazone (1.0 mg/kg) in combination with glimepiride (0.5 mg/kg) or with pioglitazone (2.5 mg/kg) in combination with glimepiride (0.5 mg/kg). Diabetic nephropathy markers were evaluated by biochemical and ELISA kits and renal structural changes were examined by light microscopy and transmission electron microscopy. Results show that the combination of pioglitazone with glimepiride is more effective in amelioration of diabetic nephropathy than rosiglitazone with glimepiride drug therapy due to glycemic control, suppressing albumin excretion rate, total protein excretion rate and augmented TNF-a signaling during the development of streptozotocin induced type 2 diabetic nephropathy.

## INTRODUCTION

Diabetes is a metabolic disorder characterized by chronically elevated blood glucose level above the normal range. Two main forms of diabetes are type 1 and type 2. Type 1 is an autoimmune disorder with complete loss of β-cell function. Type 1 diabetes is insulin dependent, the so called insulin dependent diabetes mellitus (IDDM). Type 2 diabetes, also known as non-insulin dependent diabetes mellitus (NIDDM), is more prevalent and responsible for 90% of the disease[1]. Type 2 diabetes is characterized by two basic abnormalities: impairment of insulin secretion and decrease in insulin action. Chronic hyperglycemia is a major initiator of diabetic micro- and macrovascular complications. Diabetic vascular complications are the leading cause of end stage renal failure, acquired blindness, and a variety of neuropathies and cardiovascular diseases, which account for disabilities and high mortality rates in patients with diabetes. Diabetic nephropathy is one of the main complications of diabetes, developing in 25%-40% of diabetic patients and finally leading to kidney transplantation and artificial dialysis treatment within 20-25 y[2],[3],[4],[5],[6]. Results of large scale epidemiological studies such as the UK Prospective Diabetes Study and Diabetes Control and Complications Trial indicated that hyperglycemia is the main cause of diabetic complications, including diabetic nephropathy[7],[8].

Glucose dependent pathways are activated within the diabetic kidney. These include increased oxidative stress, renal polyol formation, accumulation of advanced glycated end-products and secretion of pre-sclerotic cytokines, such as transforming growth factor-β1 (TGF-β1). These pathways ultimately lead to increase in renal albumin permeability and extra cellular matrix accumulation, which results in increasing proteinuria, glomerulosclerosis and tubulointerstitial fibrosis[9]. Diabetic nephropathy (DN) is a major cause of end-stage renal disease (ESRD) in the general population. It is estimated that DN will develop in about 40% of all patients with diabetes; therefore, prevention is critical for delaying the development and progression of diabetic kidney disease[10],[11],[12]. Once overt DN is present, ESRD can be postponed, but usually not prevented, even by effective antihypertensive treatment and careful glycaemic control[13],[14]. Both peroxisome proliferator activated receptor α/γ (PPAR α/γ) are expressed in the kidney, and their agonists exhibit renoprotective effects in type 2 diabetes[15]. Thiazolidinediones (TZDs), the most widely used PPAR-γ agonists clinically, have become blockbuster drugs in the management of type 2 diabetes mellitus[16]. The UK Prospective Diabetes Study found that intensive treatment with sulfonylurea or insulin reduced micro-vascular complications by 15%[7]. Other investigators found that in inadequately controlled type 2 diabetic patients, the combination of sulfonylurea and TZD produces significant improvement in glycemic control and is safe and well tolerated[17].

Researchers focus current therapeutic interventions on prevention or slowing of the progression of DN in experimentally induced type 2 diabetes using a combination of second generation sulfonylurea agent (glimepiride) and TZDs (pioglitazone and rosiglitazone). In the present study, we assessed the effect of combination therapy on the progression of DN by determining albumin excretion rate[18], total protein excretion rate[19], and contents of TGF-β1[20], fibronectin[21], tumor necrosis factor-α (TNF-α)[22], and transferrin[23] and by further studying renal structural changes.

## MATERIALS AND METHODS

### Animals

The experimental protocol was approved by the Institutional Animal Ethical Committee (Protocol number: DIPSAR/IAEC/2007/12). Wistar albino rats of either sex weighing 150-250 g were procured from the animal house, DIPSAR, New Delhi, India. The animals were housed under standard laboratory conditions of (21±2)°C, and a relative humidity of 55% and a 12:12 h light: dark cycle were maintained during the study. The animals were given standard rat pellet and tap water *ad libitum*. Three female rats were caged with one male rat for mating. On the early morning of the next day, vaginal smears were checked for pregnancy. Smears showing the presence of sperms were identified as pregnant. The pregnant female rats were caged singly. After 21-23 d (gestation period), the animals that delivered pups (neonatal rats) were used for further studies.

### Induction of type 2 diabetes

Streptozotocin (Sigma-Aldrich, St. Louis, MO, USA) at a dose of 90 mg/kg, in freshly prepared citrate buffer (0.1 mol/L, pH 4.5), was injected intraperitoneally to 2-d-old neonatal rats using 26 gauge needles[24]. The injection site was the dorsal midpoint between the pelvis and ribs close to the right side of the spine. Six w after the injection of streptozotocin (STZ), blood glucose of the induced rats was estimated. Animals showing fasting blood glucose ≥150 mg/dL were considered as type 2 diabetes mellitus positive rats[25].

### Experimental groups

Rats of either sex were randomly allotted into different experimental groups for 8 w (*n* = 7 for each group). The first group was the normal control group treated orally with 0.5% carboxyl methyl cellulose (vehicle)(w/v). The second group was the diabetic control group treated orally with 0.5% carboxyl methyl cellulose (vehicle)(w/v). The third group was the diabetic group treated orally with a combination of rosiglitazone (1.0 mg/kg) and glimepiride (0.5 mg/kg). The fourth group was the diabetic group treated orally with a combination of pioglitazone (2.5 mg/kg) and glimepiride (0.5 mg/kg). The solutions of drugs were freshly prepared in 0.5% carboxyl methyl cellulose (w/v) before oral administration by an oral catheter on each morning.

### Collection of blood sample

At the end of drug treatment, all the animals were fasted overnight but allowed free access to water. On the next morning, blood sample was withdrawn by the retro orbital sinus under mild ether anesthesia. The blood samples were collected into a vacutainer, which had been precoated with EDTA as anticoagulant. Blood samples were centrifuged at 3,000 rpm for 10 min in a refrigerated centrifuge. The plasma separated as straw colored supernatant was used for various biochemical parameters. It was stored at -20°C until the completion of analysis.

### Collection of urine sample

At the end of drug treatment, all the animals were kept in metabolic cages for 24 h. Animals were fasted but allowed free access to water. Urine samples were collected after 24 h in urine collecting bottles.

### Measurement of renal function and biochemical parameters

Blood glucose was measured by Accu-Chek Active glucose strips. Albumin excretion rate and total protein excretion rate in urine were measured using Span and Ranbaxy diagnostic kits by autoanalyser (Echo, Logotech Pvt. Ltd, India). Plasma TGF-β1 (Diaclone, France), insulin (SPI Bio, USA), fibronectin (AssayPro, USA), TNF-α (Diaclone, France) and transferrin (ICL, USA) were measured by ELISA according to the instructions of the manufacturers.

### Histopathological examination

At the end of the experiments, all rats were sacrificed and pathological analysis of the kidney was performed. The kidney tissues were preserved in buffered neutral formalin and stored at -20°C until being processed for histopathological studies[26]. Tissues were preserved in 1% (w/v) glutaraldehyde and 4% (w/v) formaldehyde in phosphate buffer (pH-7.2) at 4°C until being processed for electron microscopy. Tissues were processed for histopathological studies at room temperature After processing, sections were stained using hematoxylin-eosin (H&E) stain using Harris's alum hematoxylin and stock 1% (w/v) alcohol eosin solution. The stained sections were finally mounted in D.P.X. mountant. Light microscopy (×400) was used for blinded qualitative histological analysis. Transmission electron microscopies of kidney samples of different groups were performed at the Institute of Pathology-ICMR, New Delhi, India.

### Statistical analysis

The results were shown as mean±SEM. To analyze differences in variables before and after treatment, paired Student's *t*-test was used. Comparison between different groups was done using one-way ANOVA followed by Student-Newman-Keuls Method. *P* values < 0.05 were considered statistically significant. Statistical analysis was done using Sigma Stat 3.5.

## RESULTS

### Fasting blood glucose

Blood glucose level was significantly increased in the diabetic group as compared to the control group (***Table 1***). Diabetic animals treated with the combination therapy of rosiglitazone with glimepiride and pioglitazone with glimepiride showed significant decrease in fasting blood glucose levels as compared with those before the drug treatment.

**Table 1:** Changes in fasting blood glucose in studied groups

Group Presentation	Fasting blood glucose (mg/dL)	Fasting blood glucose (mg/dL)
	Before treatment	After treatment
N	80.0 ± 2.5	102.0 ± 2.5
D	192.0 ± 2.5	171.0 ± 2.5
P + G	183.0 ± 7.0	100.0±5.5**
R + G	169.0 ± 8.0	108.0 ± 4**

N: Normal control; D: Diabetic control; P+G: Pioglitazone + Glimepiride; R+G: Rosiglitazone + Glimepiride. **P < 0.01 compared with before treatment.

(mean±SEM, *n* = 6)

### Albumin excretion rate in urine

Albumin excretion rate was significantly increased in the diabetic group as compared to the normal control group (***Table 2***). There was a significant decrease in albumin excretion rate in the treated group compared to the diabetic group. Additionally, more significant decrease was found in the pioglitazone and glimepiride combination group compared with the rosiglitazone and glimepiride combination group.

### Total protein excretion rate in urine

Total protein excretion rate was significantly increased in diabetic group as compared to normal control group (***Table 2***). There was a significant decrease in total protein excretion rate in the treated group compared to the diabetic group. Additionally, more significant decrease was found in the pioglitazone and glimepiride combination treatment group compared with the rosiglitazone and glimepiride combination treatment group.

**Table 2:** Changes in albumin and total protein excretion rate (mg/d) in rats receiving different regiments

Group	Albumin Excretion	Total Protein Excretion
	Rate (mg/d)	Rate (mg/d)
N	47.96±8.09	201.10±16.20
D	83.43±4.55^##^	358.23±15.41^###^
P+G	32.91±6.81**	249.05±7.53**
R+G	54.40±15.23^	306.06±15.94^

N: Normal control; D: Diabetic control; P+G: pioglitazone + glimepiride; R+G: rosiglitazone + glimepiride. ^##^*P* < 0.01 *vs* N, ^###^*P* < 0.001 *vs* N, ˆ*P* < 0.05 *vs* D, **P < 0.01 *vs* D.

(mean±SEM; *n* = 6)

### Plasma fibronectin

Plasma fibronectin was significantly increased in the diabetic group as compared to the normal control group (***Table 3***). There was a significant decrease in plasma fibronectin levels in the pioglitazone and glimepiride combination group compared to the diabetic group.

### Plasma TGF-β1

Plasma TGF-β1was significantly increased in the diabetic group as compared to the normal control group (***Table 3***). There was a significant decrease in plasma TGF-β1 content in the treated group compared to the diabetic group.

### Plasma TNF-α

Plasma TNF-α content was significantly increased in the diabetic group as compared to the normal control group (***Table 3***). There was a significant decrease in plasma TNF-α in the treated group compared to the diabetic group. Additionally, more significant decrease was found in the pioglitazone and glimepiride combination treatment group compared with the rosiglitazone and glimepiride combination treatment group.

**Table 3:** Changes in plasma fibronectin, transforming growth factor beta-1 and tumor necrosis factor alpha in rats receiving different regiments

Groups	Fibronectin	TGF Beta	TNFα
	(mg/mL)	(ng/mL)	(pg/mL)
N	1.374±0.088	5.50±1.32	14.81±0.86
D	1.725±0.079^#^	38.63±3.76^###^	23.02±3.40^###^
P+G	1.149±0.057*	03.70±1.20***	10.53±0.40***
R+G	1.678±0.235	8.60±2.56***	18.37±1.47**

N: Normal control; D: Diabetic control; P+G: pioglitazone + glimepiride; R+G: rosiglitazone + glimepiride. ^#^*P* < 0.05 *vs* N, ^###^*P* < 0.001 *vs* N, *P < 0.05 *vs* D, **P < 0.01 *vs* D, ***P < 0.001 *vs* D.

(mean±SEM, *n* = 6)

### Plasma transferrin

Plasma transferrin content was significantly increased in the diabetic group as compared to the normal control group (***Table 4***). There was a significant decrease in plasma transferrin in the treated group compared to the diabetic group.

### Fasting plasma insulin

Fasting plasma insulin levels were significantly increased in the diabetic group as compared to the normal control group (***Table 4***). There was a significant decrease in fasting plasma insulin levels in the treated group compared to the diabetic group.

**Table 4:** Changes in plasma transferrin and fasting plasma insulin in rats receiving different regiments

Groups	Transferrin	Fasting plasma
	(mg/mL)	insulin (ng/mL)
N	1.799±0.016	0.550±0.100
D	2.045±0.023^#^	1.503±0.119^###^
P+G	1.480±0.090*	0.140±0.088***
R+G	1.514±0.039*	0.525±0.099***

N: Normal control; D: Diabetic control; P+G: pioglitazone + glimepiride; R+G: rosiglitazone + glimepiride. ^#^*P* < 0.05 *vs* N, ^###^*P* < 0.001 *vs* N, *P < 0.05 *vs* D, ***P < 0.001 *vs* D.

(mean±SEM, *n* = 6)

### Histopathological study in rats receiving different regiments

**Fig. 1:**
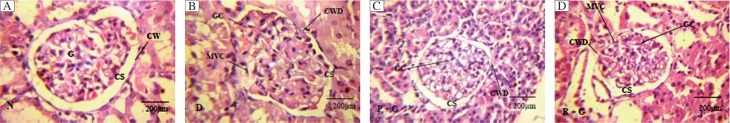
Light microscopic findings of glomeruli in Wistar albino rats (H & E staining, ×400). A: The normal group (N). B: The diabetic group (D). C: The pioglitazone and glimepiride combination treatment diabetic group (P+G). D: The rosiglitazone and glimepiride combination treatment diabetic group (R+G). In the diabetic group, CWD-capsular wall distortion, GC-glomerular condensation, MVC-micro vascular condensation and decrease in CS-capsular space were observed. But in the treatment groups [(P+G) and (R+G) groups], these changes were attenuated.

**Fig. 2:**
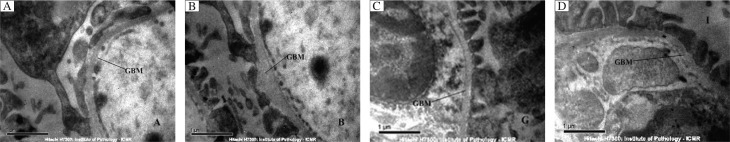
Transmission electron micrograph of representative glomerular capillary loop from Wistar albino rats (Magnification×30,000). A: The normal group with vehicle treatment (N). B: The diabetic group (D). C: The pioglitazone and glimepiride combination treatment diabetic group (P+G). D: The rosiglitazone and glimepiride (R+G) combination treatment diabetic group. GBM: glomerular basement membrane.

**Fig. 3:**
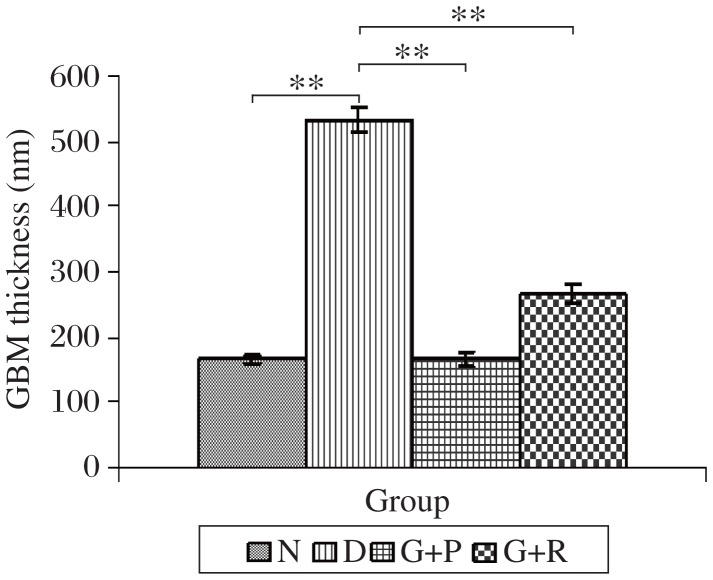
GBM thickness (nm) analyzed after drug treatment of different groups. N: Normal control. D: Diabetic Control. P+G: pioglitazone and glimepiride. R+G: rosiglitazone and glimepiride. values are mean±SEM, *n*=6, **P < 0.001.

Light microscopy study in H&E stained kidney tissue sections revealed greater capsular wall distortion, glomerular condensation, micro-vascular condensation and decrease in capsular space in the diabetic group than the normal control group (***Fig. 1A*** and ***1B***). But in the treatment groups, these changes were attenuated. The glimeperide and pioglitazone combination treatment group (***Fig. 1C***) showed maximum renoprotection compared to the glimepiride and rosiglitazone combination treatment group (***Fig. 1D***) due to the absence of micro-vascular condensation and improvement of capsular wall and capsular space. Transmission electron micrographs of glomerular capillary loops from the Wistar albino rats (15 w after induction of control vehicle or diabetes) of different groups were analyzed after drug treatment. In the diabetic group (***Fig. 2B***), increased glomerular basement membrane (GBM) thickness was observed as compared to the normal group (***Fig. 2A***), but in the treatment groups these changes were attenuated. The glimeperide and pioglitazone combination treatment group (***Fig. 2C***) showed maximum renoprotection as compared to the glimeperide and rosiglitazone combination treatment group (***Fig. 2D***) due to maximum decrease in GBM thickness (***Fig. 3***).

## DISCUSSION

Our study demonstrated that treatment with pioglitazone and glimepiride significantly decreased fasting blood glucose and plasma insulin. The anti-diabetic activity of glimepiride is seen because it improves insulin secretion and peripheral insulin sensitivity[27],[28]. TZD (pioglitazone and rosiglitazone) are a novel class of anti-diabetic drugs belonging to selective agonist for nuclear PPAR-γ. The antidiabetic activity of pioglitazone is seen due to the activation of genes regulating insulin sensitizing action[29]. Pioglitazone increased insulin sensitivity in part by activating kinase of the receptors through indirect effect on insulin receptors and that the drug may have useful benefits in insulin resistance of type 2 diabetes[30]. Diabetic rats treated with pioglitazone and glimepiride showed reduction in albumin excretion rate, total protein excretion rate, plasma fibronectin, TGF-β1, TNF-α, transferrin concentration and renal structural changes. Interventions that have ameliorated the progression of diabetic nephropathy have been associated with a reduction in urinary protein excretion[31], and thus renoprotective therapy should aim to achieve the maximal antialbuminuric effect[32],[33]. There are several mechanisms whereby increased plasma TGF-β1 has been shown to play a role in the pathogenesis of renal diseases[34],[35]. Thus, the reduction in plasma TGF-β1 concentration with pioglitazone and glimepiride demonstrated renoprotective effect. Pro-inflammatory cytokines such as TNF-α may play a significant role in the development of renal injury in type 2 diabetes[36],[37],[38]. Therefore, results from experimental studies indicate that inhibition of TNF-α activity is associated with beneficial renal effects, suggesting that modulation of this cytokine may have a real clinical application for the treatment of DN. Reduction in plasma fibronectin and transferrin concentration with pioglitazone and glimepiride demonstrated renoprotective effect. Glimepiride and pioglitazone combination treatment showed renoprotection due to the absence of micro-vascular condensation and attenuation of capsular wall, capsular space and glomerular basement membrane changes.

Diabetic rats treated with rosiglitazone and glimepiride showed reduction in fasting blood glucose, albumin excretion rate, total protein excretion rate, plasma insulin, TGF-β1, TNF-α, transferrin concentration and renal structural changes. Therefore, results from experimental studies indicate that rosiglitazone and glimepiride treatment produced reduction in albumin excretion rate, total protein excretion rate, plasma TNF-α concentration and renal structural changes less than treatment with pioglitazone and glimepiride combination.

The present work compared dual therapy of rosiglitazone with glimepiride versus pioglitazone with glimepiride on STZ induced type 2 DN in rats. In conclusion, our results show that the combination of pioglitazone with glimepiride is more effective in amelioration of DN than rosiglitazone with glimepiride drug therapy due to glycemic control, suppressing albumin excretion rate, total protein excretion rate and augmenting TNF-α signaling during the development of STZ induced type 2 diabetic nephropathy.
